# Post-stimulus endogenous and exogenous oscillations are differentially modulated by task difficulty

**DOI:** 10.3389/fnhum.2013.00009

**Published:** 2013-01-31

**Authors:** Yun Li, Bin Lou, Xiaorong Gao, Paul Sajda

**Affiliations:** ^1^Department of Biomedical Engineering, Tsinghua UniversityBeijing, China; ^2^Department of Biomedical Engineering, Columbia UniversityNew York, NY, USA

**Keywords:** alpha oscillations, face perception, electroencephalography (EEG), SSVEP, attention, perceptual decision-making

## Abstract

We investigate the modulation of post-stimulus endogenous and exogenous oscillations when a visual discrimination is made more difficult. We use exogenous frequency tagging to induce steady-state visually evoked potentials (SSVEP) while subjects perform a face-car discrimination task, the difficulty of which varies on a trial-to-trial basis by varying the noise (phase coherence) in the image. We simultaneously analyze amplitude modulations of the SSVEP and endogenous alpha activity as a function of task difficulty. SSVEP modulation can be viewed as a neural marker of attention toward/away from the primary task, while modulation of post-stimulus alpha is closely related to cortical information processing. We find that as the task becomes more difficult, the amplitude of SSVEP decreases significantly, approximately 250–450 ms post-stimulus. Significant changes in endogenous alpha amplitude follow SSVEP modulation, occurring at approximately 400–700 ms post-stimulus and, unlike the SSVEP, the alpha amplitude is increasingly suppressed as the task becomes less difficult. Our results demonstrate simultaneous measurement of endogenous and exogenous oscillations that are modulated by task difficulty, and that the specific timing of these modulations likely reflects underlying information processing flow during perceptual decision-making.

## Introduction

Neural oscillations, for example measured via electroencephalography (EEG), have been studied for decades in an effort to link brain state to perceptual and cognitive processing. Endogenous oscillations are attributable to internal neural processes and include a well-known set of frequencies ranging from the low delta to the high gamma band (Niedermeyer and Lopes da Silva, 1993). Exogenous oscillations are driven by the rhythms of external stimuli and are typically associated with sensory systems [e.g., steady state visually evoked potentials (SSVEP) and auditory steady state response (ASSR)] (Picton et al., 2003; Vialatte et al., 2010 review).

A prominent endogenous brain rhythm is the alpha oscillation which has been extensively investigated within the context of both pre-stimulus and post-stimulus effects (Babiloni et al., 2006; Hanslmayr et al., 2007; Freunberger et al., 2008; van Dijk et al., 2008). The neural mechanism underlying alpha oscillations also have been deeply explored (Lopes da Silva, 1991). Though alpha activity is often thought to represent an idling or inattentive state (Pfurtscheller et al., 1996 review), some studies suggest that it also reflects a suppression mechanism of irrelevant information (Worden et al., 2000; Kelly et al., 2006; Rihs et al., 2007; Foxe and Snyder, 2011; Gomez-Ramirez et al., 2011), and/or an inhibition of information processing (Klimesch et al., 1997, 2007 review). Specifically, previous studies of post-stimulus alpha activity within the context of visual object recognition have shown that alpha desynchronization was greater for the recognition of meaningful objects than it was for meaningless objects (Klimesch et al., 1997; Vanni et al., 1997; Mima et al., 2001; Freunberger et al., 2008), suggesting that post-stimulus alpha activity is related to semantic information processing.

SSVEP, an oscillatory brain response evoked by a flickering visual stimulus, is an exogenous form of frequency tagging that has been shown to index the allocation of cognitive resources such as attention (Vialatte et al., 2010 review). Many studies have reported that SSVEP amplitude is decreased when attention must compete or be split between the flicker and a background picture (Müller et al., 2008, 2011; Attar et al., 2010). Moreover, a study by Andersen and Müller in 2010 revealed that the facilitation of SSVEP amplitude for the attended stimulus is accompanied by suppression for the unattended stimulus (Andersen and Müller, 2010). These findings suggested that SSVEPs can be used as neural marker of the time course of attentional resource competition.

In this paper, we aim to simultaneously investigate how post-stimulus endogenous and exogenous oscillations are affected as a function of task difficulty during a face/car discrimination task. Specifically, we superimpose a flickering stimulus of 15 Hz upon a sequence of images and simultaneously analyze the time course and spatial distribution of exogenously-induced SSVEPs and endogenous alpha oscillations as a function of image phase coherence. Our results demonstrate that the phase coherence of the stimulus, being our surrogate for the difficulty of the visual discrimination, differentially modulates exogenously-induced SSVEPs and endogenous alpha oscillations at different times and that this may reflect underlying information processing flow during the visual discrimination task.

## Materials and methods

### Subjects

Eleven right-handed subjects (three females and eight males; mean ± SD age, 26.5 ± 5.9 years) with normal or corrected-to-normal vision participated in this study. Informed consent in accordance with the guidelines and approval of the Columbia University Institutional Review Board was obtained from all subjects.

### Stimuli and experimental procedure

**Figure 1:**
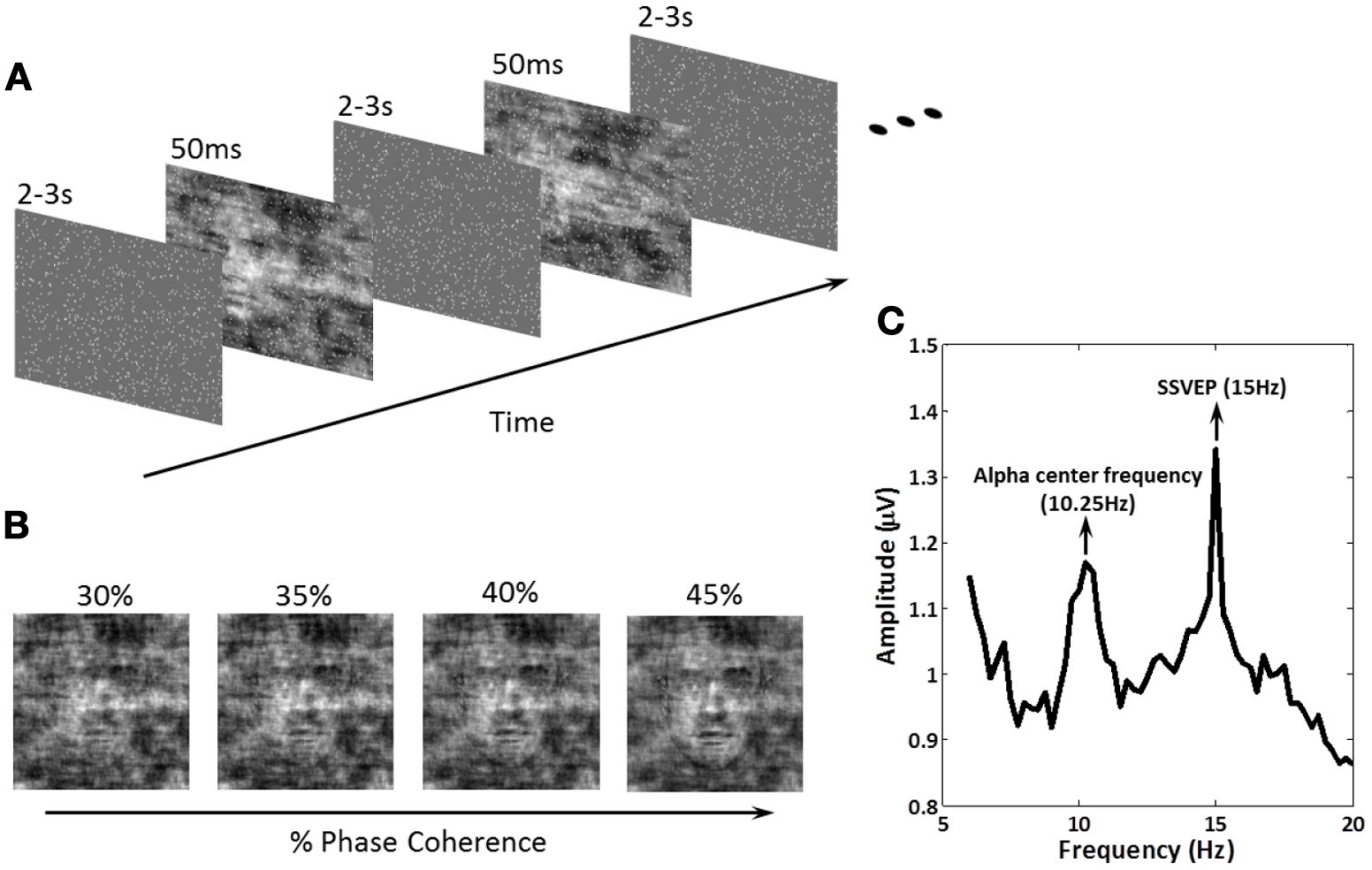
**Schematic representation of the experimental paradigm, the methodology for effecting decision difficulty and an example amplitude spectrum showing exogenous and endogenous frequencies of interest. (A)** Within a block of trials, subjects were instructed to fixate on the center of the screen. A series of face and car images at four phase coherence levels were shown in random order. Each image was presented for 50 ms followed by an inter-stimulus-interval (ISI), randomized in the range of 2000–3000 ms. Subjects were instructed to discriminate, as fast as possible, the image class (face or car) via a keyboard response. 15 Hz flickering dots were superimposed across the images for the entire experiment. Image onsets and the flickering dot pattern were not phased-locked. **(B)** Decision difficulty was controlled by changing the phase coherence in the face/car images (see “Materials and Methods” for details). Shown is an example of one of the images at the four different phase coherence levels (30, 35, 40, and 45%) used in the experiment. **(C)** The amplitude spectrum of EEG at electrode PO8 obtained by Fourier analysis for one subject. The endogenous oscillation was at the alpha frequency for the individual subject, which in this case was 10.25 Hz, while the exogenous oscillation at 15 Hz was the SSVEP produced by the flickering dots. Modulation of these amplitudes during the experiment was the focus of our analysis.

Stimuli were presented in the center of a 17′ LCD monitor with a refresh rate of 60 Hz. A set of 17 face (Max Planck Institute face database; Troje and Bülthoff, 1996) and 17 car grayscale images (image size, 512 × 512 pixels; 8 bits/pixel) were used. The car image database was the same used in Philiastides and Sajda (2006) and was constructed by taking images from the internet, segmenting the car from the background in each, converting the image to grayscale and then resizing it to be comparable in size to the face images. The pose of the faces and cars was also matched across the entire database and was sampled at random (left, right, center). Equal luminance and contrast were used. All images had identical magnitude spectra (average magnitude spectrum of all images in the database), and their corresponding phase spectra were manipulated using the weighted mean phase (WMP) (Dakin et al., 2002) technique to generate a set of images characterized by their percentage of phase coherence. In this experiment, four different phase coherence levels (30, 35, 40, and 45%) were utilized to create tasks of different difficulty levels (Figure 1B).

The schematic of the experimental design is shown in Figure 1A. Face/car (i.e., “task”) images were presented for 50 ms followed by a random inter-stimulus interval (ISI) uniformly distributed between 2000 and 3000 ms. During the ISI, a uniform grayscale image was presented as the background, which had the same size and average grayscale value as the task images. Frequency tagging was done by superimposing a total of 900 randomly-placed small white squares (each 3 × 3 pixels) on the stream of images (tagging was continuous across the task images and ISI), with the white squares having a flicker frequency of 15 Hz. 15 Hz was chosen so that endogenous alpha power could also be measured. The ISIs was randomly generated after each trial, and task images were not phase-locked to the 15 Hz flicker, which effectively reduced the interaction between the event related potentials (ERPs) and SSVEPs.

Subjects were instructed to discriminate task images as rapidly as possible. Each experimental block consisted of 34 trials of face and car images at each of four different phase coherence levels, with images presented randomly within a block. In each experiment, there were six blocks in total. When a task image appeared, subjects reported their decision regarding the category of the image by pressing a button on the keyboard, with the left arrow key for cars and right arrow key for faces, using one of two fingers on their right hands. Subjects were instructed to maintain central fixation throughout the entire experiment and to respond as quickly and accurately as possible.

### Data acquisition

Participants were seated in an electrostatically shielded room and positioned at a 1 m distance from the screen. EEG was recorded using a Sensorium 84-channel Ag/AgCl electrode system (Sensorium Inc., Vermont, USA). The ground channel was located between the eyebrows, and all channels were referenced to the left mastoid. All impedances were below 20 kΩ and the sampling rate was 1000 Hz. Stimulus events and motor responses were recorded on separate channels.

### Data processing

Epochs were extracted according to the task events. Trials with strong eye movements or other movement artifacts were manually rejected, resulting in less than 20% trials rejected. Only EEG from correct trials with reaction times below 1000 ms was analyzed. During the behavioral and EEG data analysis, face and car trials at the same coherence level were considered equal in difficulty. In other words, there were only four difficulty conditions, corresponding to the four phase coherence levels.

For every subject, decision accuracy and mean reaction time from correct trials at each phase coherence level were calculated. To test the consistency of behavioral performance across subjects, a balanced One-Way ANOVA, testing the effect of phase coherence levels (30, 35, 40, and 45%), was performed. Also, paired *t*-tests were performed between each pair of phase coherence levels.

Since SSVEP is primarily seen in visual cortex (Regan, 1989; Vialatte et al., 2010 review) and alpha oscillations were predominantly found at parietal-occipital areas (Adrian and Matthews, 1934; Palva and Palva, 2007), we re-referenced the EEG to electrode Fz since it is distant from visual cortex. The amplitude spectrum of EEG waveforms at electrode PO8 obtained by Fourier analysis (Figure 1C) illustrates the SSVEP at 15 Hz relative to the alpha power. We confirmed that each subject's central alpha frequency is well separated from the exogenous oscillation at 15 Hz induced by the flickering stimuli.

The time course of SSVEP amplitude at each phase coherence level was quantified by the following steps.

1. Narrow band pass filtering was done using a zero-phase filter within the range of 15 ± 1.6 Hz to isolate the SSVEP signal.
2. Calculating the analytic signal of the filtered EEG by applying the Hilbert transform.
3. Estimating the instantaneous SSVEP amplitude from the complex amplitude of the analytic signal.
4. Normalizing the instantaneous SSVEP amplitude by subtracting the averaged amplitude of a baseline from −200 to 0 ms before the target and then dividing by the same baseline (Andersen and Müller, 2010).

Specially, we assumed the baselines of all conditions are the same, and therefore only calculated one baseline for each subject by averaging all trials across all conditions. The result is the normalized instantaneous SSVEP amplitude reflecting the changes in SSVEP amplitude relative to the baseline, which ensures that each subject contributes, more or less, equally to the average, avoiding the group results from being dominated by a single subject.

**Figure 2:**
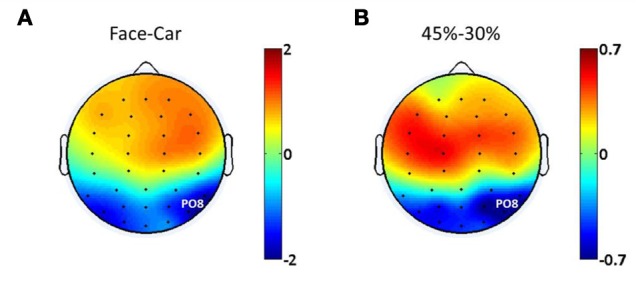
**Spatial distributions of ERP amplitude differences between (A) face and car trials at 170 ms post-stimulus, (B) phase coherence levels of 45 and 30% at 220 ms post-stimulus**.

Evidence from EEG, magnetoencephalography (MEG) and fMRI suggests the existence of face discriminating activity in right lateral occipital cortex (rLOC, near electrode PO8) (Bentin et al., 1996; Jeffreys, 1996; Kanwisher et al., 1997; Liu et al., 2000), while a study by Philiastides and Sajda (2007) demonstrated a connection between the LOC and decision difficulty. To identify electrodes that were most relevant to both discrimination and difficulty in the task, we analyzed the spatial distributions of the ERP amplitude differences at 170 ms post-stimulus between face and car trials at the 45% phase coherence level (Figure 2A), as well as at 220 ms post-stimulus comparing the 30 and 45% phase coherence levels (Figure 2B). This approach was taken since previous studies have demonstrated that the difference between categories was characterized by the amplitude difference of the N170 component, while the difficulty effect was quantified by the amplitude difference of the D220 component (Philiastides and Sajda, 2006; Philiastides et al., 2006). Our primary analysis of endogenous and exogenous frequency modulations was then done on the electrode with the most significant selectivity for face vs. car and task difficulty (i.e., sensitivity to phase coherence level). This turned out to be electrode PO8. Additional analysis showing the spatial distribution of the modulations across all electrodes is reported in Figures 5 and 7.

To identify time periods in which phase coherence had a significant effect, we performed a set of statistical tests on the normalized SSVEP amplitude. First, paired *t*-tests between phase coherence levels of 30 and 45% were conducted over each time point from 0 to 800 ms post-stimulus on all electrodes. Next, we adopted a modified cluster-analysis approach to correct for multiple comparisons and identify time periods within which the SSVEP amplitude between phase coherence levels was significantly different. Specifically, the time points over which the null hypothesis was rejected at a significance level of 0.05 were selected and clustered based on their temporal adjacency. The maximum temporal period across all contiguous clusters were used as for constructing the cluster-level statistics. The data were then randomized across two phase coherence levels (30 and 45%) to generate shuffled cluster-level statistics. We performed all possible permutations of the 11 subjects to generate the shuffled cluster-level statistics. Finally, corrected *p*-values were calculated by comparing the values of the cluster-level statistics of the original data against the distribution of the shuffled cluster-level statistics across permutations (Maris and Oostenveld, 2007; Rohenkohl and Nobre, 2011). Subsequently, the time periods with corrected *p*-values less than 0.05 at electrode PO8 were selected for analyzing the spatial distribution of SSVEP amplitude in parietal and occipital areas at each phase coherence level.

The effect of phase coherence on the alpha band was quantified using the same processing steps as the SSVEP analysis, described above, except that the filtering band was specific for each subject, in terms of their alpha center frequency ±1.6 Hz. The mean alpha center frequency across all subjects was 10.5 Hz (SD, 1.0 Hz). Since motor related oscillations (mu rhythm) share a common frequency band (8–12 Hz) with endogenous alpha oscillations, response-locked data of both SSVEP and alpha oscillations were also analyzed to investigate the effect of the motor response.

## Results

### Behavioral results

**Figure 3:**
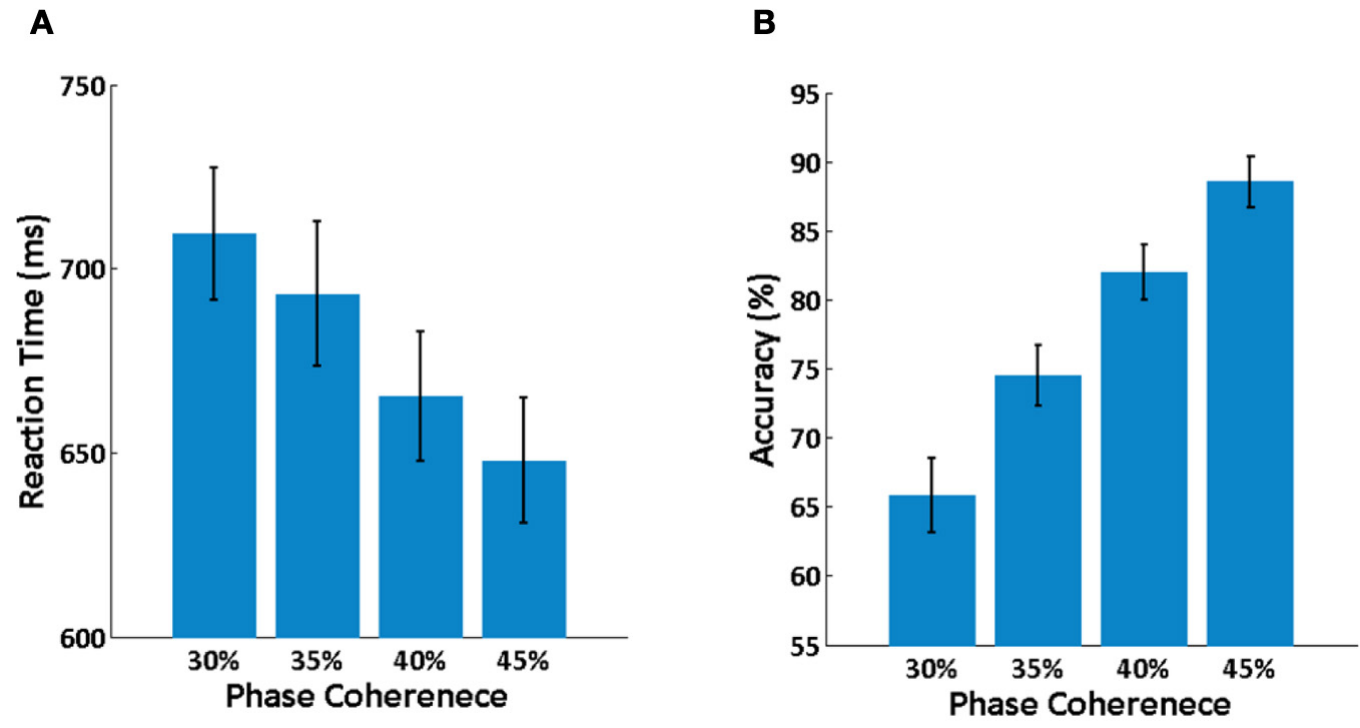
**Behavioral results. (A)** Mean reaction time averaged over subjects at each phase coherence level. **(B)** Average decision accuracy across subjects at each phase coherence level. Error bars indicate the standard error.

Averaged behavioral performance across all subjects at each phase coherence level is shown in Figure 3. It is clear that reaction time is increased (Figure 3A) and decision accuracy is decreased (Figure 3B) as phase coherence level decreases. A balanced One-Way ANOVA demonstrates a significant effect of phase coherence level on reaction time [*F*_(3, 36)_ = 2.94, *p* < 0.05] and decision accuracy [*F*_(3, 40)_= 17.93, *p* < 0.001]. A set of paired *t*-tests demonstrates that there is a significant difference between any two phase coherence levels for reaction time (*p* < 0.01) and decision accuracy (*p* < 0.01). Behavioral results thus clearly demonstrate a significant effect of phase coherence on decision difficulty as measured via reaction time and accuracy.

### Effect of phase coherence on exogenous oscillations

**Figure 4:**
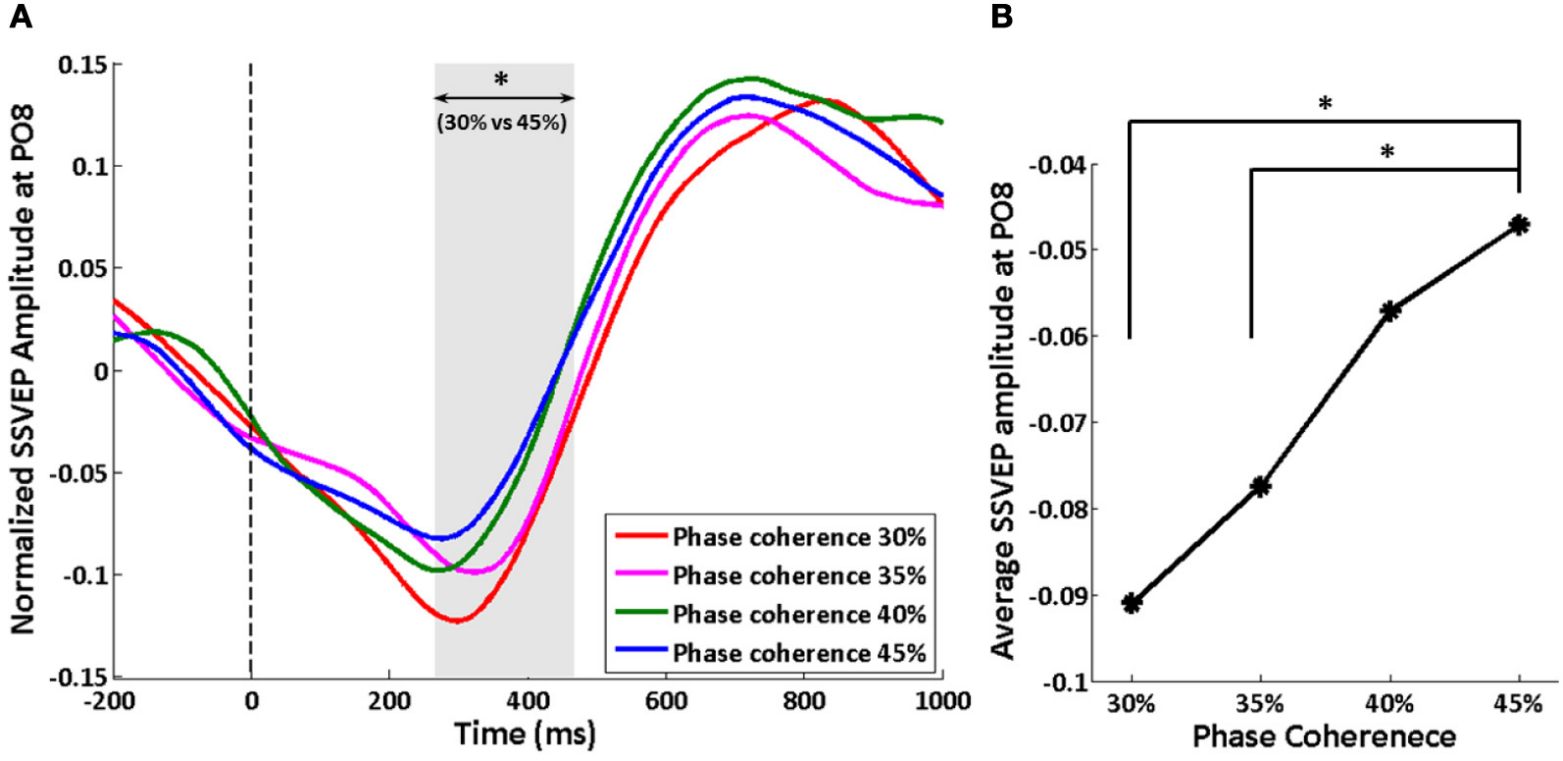
**Effect of decision difficulty on exogenous oscillations as measured by normalized SSVEP amplitudes at electrode PO8. (A)** Time course of normalized SSVEP amplitude, shown for each of four phase coherence levels. The shaded area indicates the time period (266–466 ms) having a significant difference in normalized SSVEP amplitude between phase coherence levels of 30 and 45% as assessed by paired *t*-test across subjects and cluster-level statistics (*p* < 0.05). The vertical dashed line indicates the onset of task images. **(B)** Average SSVEP amplitude from 266 to 466 ms at each phase coherence level. Asterisks indicate significant differences (paired *t*-test, *p* < 0.05).

We tracked the time course of the SSVEP amplitude as a way to explore how exogenous oscillations are modulated by phase coherence and therefore decision difficulty. Figure 4A shows the time course of the normalized SSVEP amplitude at electrode PO8. There is a suppression of normalized SSVEP amplitude immediately after stimulus onset. Using a paired *t*-test between phase coherence levels of 30 and 45%, a significant effect of phase coherence (*p* < 0.05) was observed from roughly 266 to 466 ms post-stimulus. This time period was confirmed to be significant with multiple comparisons correction using cluster-level statistics (*p* < 0.05). The average SSVEP amplitude in this time period at electrode PO8 for each of the four phase coherences is presented in Figure 4B. As phase coherence increases, the average SSVEP amplitude also increases, indicating a greater suppression of SSVEP amplitude at lower phase coherence levels. The average SSVEP amplitudes in this period at phase coherence levels of 30 and 35% are significantly different from those at a phase coherence level of 45% (paired *t*-test between 30 and 45% phase coherence levels: *t*_(10)_ = 3.16, *p* = 0.010; paired *t*-test between 35 and 45% phase coherence levels: *t*_(10)_ = 2.63, *p* = 0.025).

**Figure 5:**
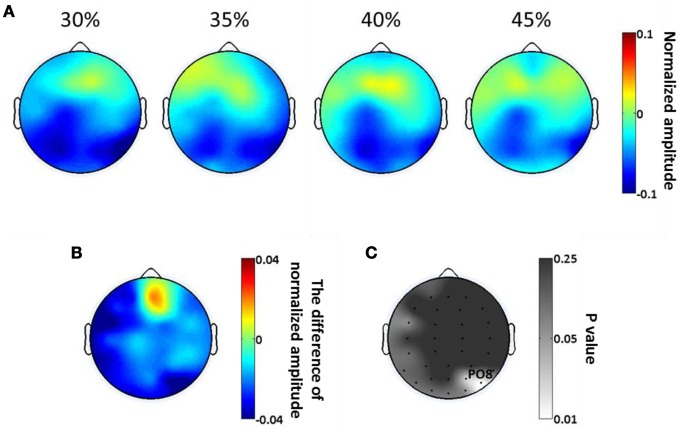
**Spatial distribution of exogenous oscillatory modulations, within a 266–466 ms time window, as a function of decision difficulty. (A)** Scalp topographies showing the scalp distribution of average SSVEP amplitude for the four phase coherence (i.e., difficulty) levels. **(B)** The average difference in SSVEP amplitude between phase coherence levels of 30 and 45% (30–45%). **(C)** The *p*-values at each electrode location, assessing the average SSVEP amplitudes via a paired *t*-test between phase coherence levels of 30 and 45%. Lower *p*-values indicate more significant differences in mean SSVEP amplitude modulations between the 30 and 45% phase coherence levels.

The scalp topologies of the average SSVEP amplitude from 266 to 466 ms, for each of the four phase coherence levels, are plotted in Figure 5A. The reduction of SSVEP power is mainly in occipital areas, and this reduction is greater for lower phase coherences. The spatial distribution of the difference in SSVEP amplitude between 30 and 45% coherence levels (Figure 5B) illustrates that phase coherence/task difficulty effects are substantial in the region near electrode PO8. The *p*-value at each electrode location, as assessed by a paired *t*-test, is plotted in Figure 5C. In occipital areas, only electrodes around PO8 show significant effects (*p* < 0.05) of phase coherence/task difficulty.

### Effect of phase coherence on endogenous oscillations

**Figure 6:**
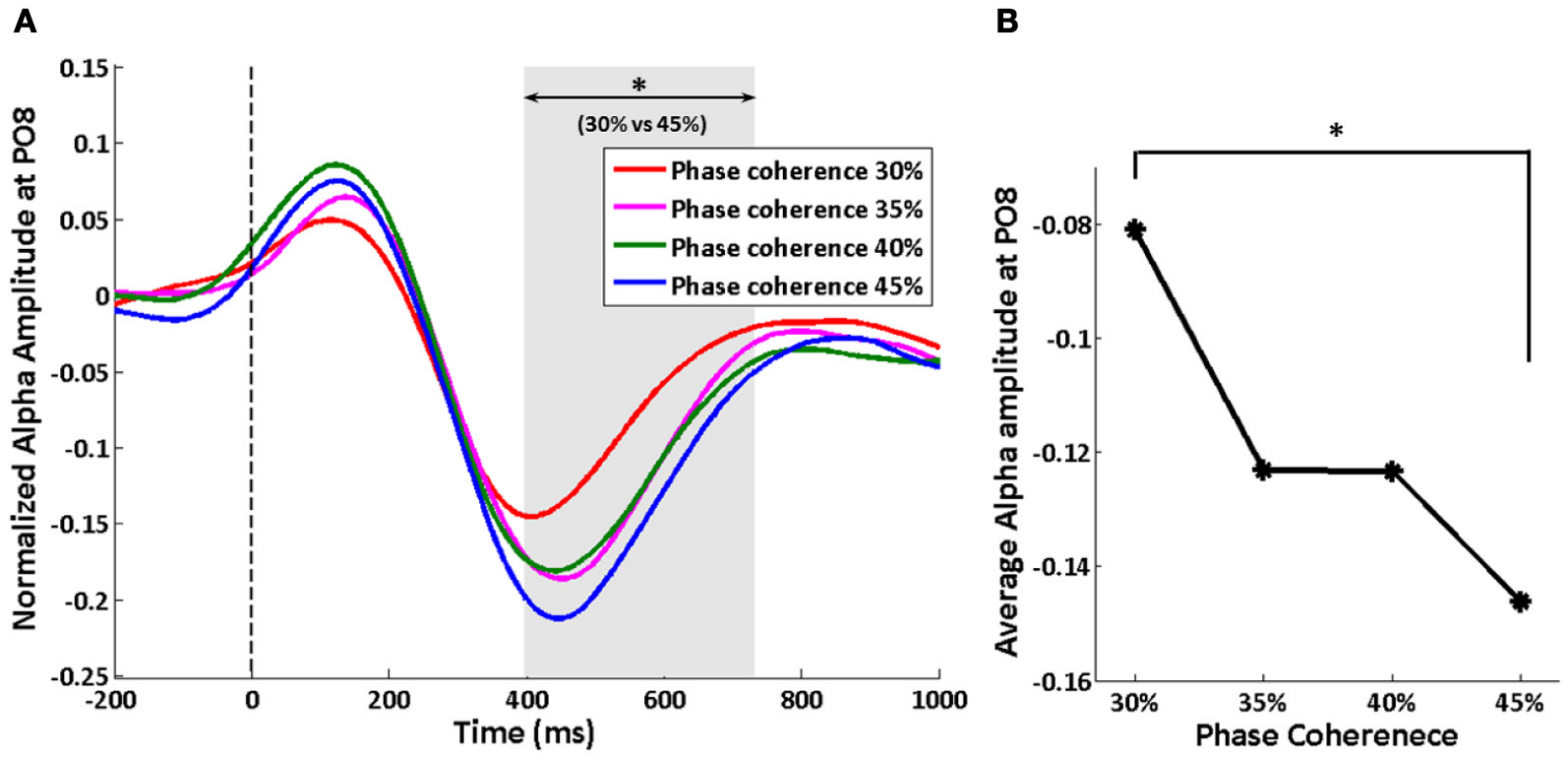
**Effect of decision difficulty on endogenous oscillations as measured by normalized alpha amplitude at electrode PO8. (A)** Time courses of normalized alpha amplitude, shown for each of four phase coherence levels. The shaded area indicates the time period (397–731 ms) having a significant difference in normalized alpha amplitudes between phase coherence levels of 30 and 45% as assessed by paired *t*-test across subjects and cluster-level statistics (*p* < 0.05). The vertical dashed line indicates the onset of task images. **(B)** The average alpha amplitude from 397 to 731 ms at each phase coherence level. Asterisks indicate significant differences (paired *t*-test, *p* < 0.05).

Changes in normalized endogenous alpha oscillations at electrode PO8 are shown in Figure 6A. The alpha amplitude first increase in the 250 ms time period after the stimulus, and then falls below the baseline. However, only the time period from 397 to 731 ms shows a significant difference between phase coherence levels of 30 and 45%, as assessed by a paired *t*-test (*p* < 0.05) and cluster-level statistics (*p* < 0.05). The average suppression in this time period increases with increasing phase coherence levels, which is the reverse of the difficulty modulation on exogenous oscillations (Figure 6B). A significant difference was only found between 30 and 45% phase coherence levels (paired *t*-test: *t*_(10)_ = 3.36, *p* = 0.007).

**Figure 7:**
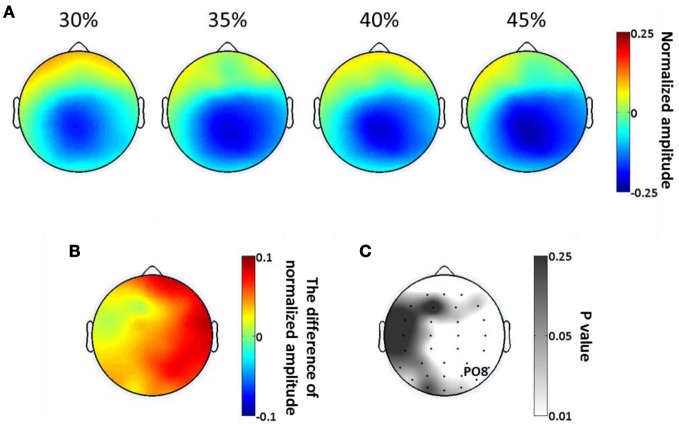
**Spatial distribution of endogenous oscillatory modulations, within 397–731 ms time window, as a function of decision difficulty. (A)** Scalp topographies showing the scalp distribution of average alpha amplitude for the four phase coherence (i.e., difficulty) levels. **(B)** The power difference of average alpha amplitude between phase coherence levels of 30 and 45% (30–45%). **(C)** The *p*-values at each electrode location, assessing the average alpha amplitudes via a paired *t*-test between phase coherence levels of 30 and 45%. Lower *p*-values indicate more significant differences in mean alpha amplitude modulations between the 30 and 45% phase coherence levels.

As in our analysis of SSVEP, we calculated the scalp distributions of average alpha amplitude in the significant time period (397–731 ms) at each phase coherence level (Figure 7A). Alpha suppression is centered in parietal and occipital regions, with suppression being greater at high phase coherence levels. By plotting the difference in alpha power between 30 and 45% phase coherence levels and the scalp maps of *p*-values, as shown in Figures 7B and C, we can see that the modulation of the alpha power by phase coherence/task difficulty appears in right lateral parietal-occipital regions.

### Effect of the motor response

**Figure 8:**
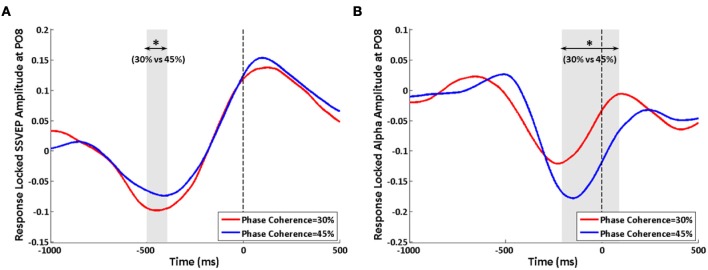
**Effects of decision difficulty on response-locked data. (A)** SSVEP and **(B)** alpha oscillations at electrode PO8. The shaded area indicates the time period that shows a significant difference in amplitudes between phase coherence levels of 30 and 45%. The vertical dashed lines were locked with reaction time. Asterisks indicate significant differences (paired *t*-test, *p* < 0.05).

When analyzing the data by aligning trials according to their reactions times, effects of decision difficulty on exogenous induced SSVEP and endogenous alpha oscillations can still be observed between the highest and lowest phase coherence levels. Similar to the stimulus-locked analysis, a lower SSVEP power for 30% phase coherence trials at electrode PO8 is observed roughly from −500 to −400 ms before the response (Figure 8A), while a reversed effect of task difficulty on endogenous alpha oscillations is seen lasting from approximately −200 ms before the response until 100 ms after the response (Figure 8B). The timing of the period that showed a significant difference between difficulty levels was consistent with results of the average reaction time and stimulus-locked analysis. However, the duration was shorter comparing with the corresponding stimulus-locked analysis.

## Discussion

The results of the present study demonstrate that task difficulty, represented here via manipulation of the phase coherence of the visual stimulus, modulates the amplitudes of exogenously induced SSVEPs and endogenous alpha oscillations in different ways and at different times. In our experiment, the amplitude modulations of SSVEP are in response to the task-irrelevant 15 Hz flickers, while endogenous alpha oscillations reflect the response of an intrinsic rhythm to the face vs. car decision. To compare the effects of task difficulty on SSVEP and endogenous alpha oscillations, we will now summarize several of our observations. First, SSVEP amplitude after the stimulus is immediately decreased, while there is a delay of 250 ms before suppression of alpha amplitude. Second, the time periods reflecting significant modulation by task difficulty are 266–466 ms and 379–731 ms for SSVEP and alpha oscillations respectively. Third, the correlation between the phase coherence level and the amplitude suppression for SSVEP is opposite to what is seen for alpha. With the increase of the phase coherence level, the reduction of SSVEP amplitude is decreased, while the reduction of alpha amplitude is increased. Fourth, the SSVEP suppression appears in occipital regions, and the difficulty effect is most significant near electrode PO8. Alpha changes, conversely, are widespread over a broader region in parietal and occipital areas though the most significant modulation of alpha suppression by decision difficulty is primarily in right lateral parietal-occipital areas.

Our findings showed that the suppression of SSVEP amplitude from 266 to 466 ms was greater at lower phase coherence levels. SSVEP can be used as an objective measure of the allocation of attentional resources (Vialatte et al., 2010 review). The facilitation and suppression of the SSVEP response to the attended and ignored stimuli, respectively, was reported in a study by Andersen and Müller (2010). Previous work using exogenous frequency tagging to investigate to what extent emotional pictures bias competition for attentional processing resources reported a larger decrease in SSVEP amplitude when visual processing load was increased (Müller et al., 2008, 2011; Attar et al., 2010). In addition, previous studies have suggested that additional attentional resources are needed to maintain decision accuracy when the task becomes more difficult (Binder et al., 2004; Grinband et al., 2006; Philiastides et al., 2006; Heekeren et al., 2008 review). Therefore, our observation that there is a greater reduction of SSVEP amplitude for difficult decisions can be interpreted as a neural correlate of this competition for attentional processing resources and supports the hypothesis of the need for more attentional resources when making a difficult decision. Furthermore, this greater SSVEP suppression at low coherence levels is consistent with the perceptual load hypothesis, which says that increased perceptual difficulty of a visual task attenuates the processing of task-irrelevant, unattended distractor stimuli (Yi et al., 2004; Lavie, 2005).

Our analysis showed that normalized SSVEP amplitudes mainly decreased in occipital regions, coinciding with areas that had strong SSVEP responses (Regan, 1989; Vialatte et al., 2010 review). We also found SSVEP suppression in electrodes in close proximity to PO8 exhibited the most significant difficulty effect in our face-car discrimination task. This is consistent with previous findings that rLOC is involved in face discrimination and related to decision difficulty for this type of task (Bentin et al., 1996; Jeffreys, 1996; Kanwisher et al., 1997; Liu et al., 2000; Philiastides and Sajda, 2007).

In our study, the suppression of alpha oscillations appeared in parietal-occipital areas, and this topographic distribution of alpha desychronization has been previously reported (Klimesch et al., 1997; Doppelmayr et al., 2002). The right hemisphere in parietal-occipital regions was significantly affected by decision difficulty (Figure 7), which is possibly related to the right hemisphere dominance for face perception (Rhodes, 1985; Luh et al., 1991; Yovel et al., 2008). Object recognition studies found that alpha desynchronization over the right lateral occipital regions exhibited maximal values (Freunberger et al., 2008) and correlated with the proportion of correct object detections (Vanni et al., 1996). Thus both the endogenous and exogenous effects yield scalp topologies that are consistent with the task, suggesting that the oscillations themselves are linked to specific cortical areas involved in processing the stimuli and making the decision.

Similarly to Klimesch et al. (2007 review), the time course of endogenous alpha oscillations in our study showed a synchronization at 250 ms post-stimulus and a desynchronization around 350–750 ms. The alpha suppression showed the most significant difficulty effect in the time period of 379–731 ms, as it occurred during the time of the late component and motor response previously identified for this type of face-car discrimination task using single-trial analysis of EEG (Philiastides et al., 2006). Previous studies on object recognition/detection showed that the magnitude of alpha desynchronization was related to semantic information processing, and that the recognition of meaningful objects elicited a larger alpha suppression than that for meaningless objects (Klimesch et al., 1997; Vanni et al., 1997; Mima et al., 2001; Freunberger et al., 2008). In our study, the stimuli at high phase coherence level contained more meaningful information than that at the low phase coherence level, since task difficulty was modulated by the percentage of phase coherence. Accordingly, the pattern of alpha suppression, i.e., larger alpha suppression at high phase coherence levels, may be related to semantic information processing. It is also worth mentioning that a study by Siegel et al. (2007, 2011) found that visual gamma oscillations increased with a decrease in task difficulty and that gamma-band enhancement was accompanied by a decrease in alpha-band activity. This finding supports our results showing that decreasing task difficulty further suppresses alpha oscillations.

In this study, the difficulty of the discrimination task was manipulated by varying the phase coherence in the images. A study by Bankó et al. (2011) proposed that the effect of phase noise on sensory processing should be dissociated from overall decision difficulty, and suggested that the difficulty component seen in Philiastides et al. (2006) was merely the noise-induced modulation of a bottom-up P2 visual component. However, (Philiastides et al., 2006) explicitly used a modified face vs. car discrimination experiment using colorized images to show that the effect was on decision difficulty since it was present even while keeping the stimulus unchanged, thus reflecting that this difficulty component clearly was also indexing top-down task related processes and was not simply a function of the sensory noise. Our results are interpretable within the context of “task-difficulty” regardless of whether it is defined bottom-up by the stimulus signal-to-noise and/or top-down by the task.

Additionally, the results from a response-locked analysis showed similar waveforms for the highest and lowest phase coherence levels regardless of the systematic latency differences due to different reaction times for the different difficulty levels (Figure 8). Though the endogenous alpha oscillations in parietal-occipital areas might include a superposition of mu rhythms due to volume conduction and the significant effect for endogenous alpha activity was widespread over a region in right lateral parietal-occipital areas (Figures 7B,C), near to the motor cortex, our results from the response-locked analysis showed a significant modulation of the endogenous oscillations by task difficulty.

A potentially alternative interpretation of our work is that the phase coherence manipulation we employ creates a diffuse partial occlusion that drives processes associated with perceptual closure. Though fragmented line drawings and not phase coherence are usually used to investigate perceptual closure (Doniger et al., 2000; Sehatpour et al., 2008), it is possible that the effects we observe are the same one might see in a perceptual closure experiment. Also intriguing is the similarity in the timing of the difficulty component at 220 ms (Philiastides et al., 2006) and the Ncl closure component at 250 ms (Doniger et al., 2000). Though the phase coherence manipulation effects behavioral accuracy and reaction time in a way that one would expect if directly affecting difficulty, further work is needed to investigate whether the manipulation is specific to the subject needing to perceptually close a stimulus for recognition or if it acts on more general processes of evidence accumulation in a noisy stimulus.

In summary, using our experimental paradigm we were able to analyze SSVEP and endogenous alpha oscillations simultaneously while subjects performed a face-car discrimination task where the difficulty of the decision was varied on a trial-to-trial basis. We found that the SSVEP reduction was positively correlated with decision difficulty, while the correlation between the suppression of endogenous alpha oscillations and decision difficulty was negative. The findings indicate that the amplitude modulation of SSVEP reflects the competition for the attentional processing resources and support the hypothesis of the need for more attentional resources when making a difficult decision, while endogenous alpha oscillations may be related to semantic information processing. Lastly, our results demonstrate that exogenous and endogenous oscillations can be simultaneously measured to track the changes in ongoing stimulus-driven and endogenous activities during visual discriminations of varying difficulty.

### Conflict of interest statement

The authors declare that the research was conducted in the absence of any commercial or financial relationships that could be construed as a potential conflict of interest.
